# Anticancer Impact of Nitric Oxide (NO) and NO Combination with SMYD-3 Inhibitor on Breast Carcinomas

**DOI:** 10.3390/diseases9040082

**Published:** 2021-11-12

**Authors:** Jenna L. Gordon, Kristin J. Hinsen, Melissa M. Reynolds, Mark A. Brown

**Affiliations:** 1 Department of Chemistry, Colorado State University, Fort Collins, CO 80521, USA; jenna.short@colostate.edu; 2 Department of Biomedical Sciences, Colorado State University, Fort Collins, CO 80521, USA; khinsen@rams.colostate.edu; 3 Department of Chemistry, Department of Chemical and Biological Engineering, School of Biomedical Chemistry, Colorado State University, Fort Collins, CO 80523, USA; 4 Department of Clinical Sciences, Colorado State University, Fort Collins, CO 80521, USA; mark.brown@colostate.edu

**Keywords:** breast cancer, nitric oxide, cell viability, colony formation, cytotoxicity, apoptosis

## Abstract

Despite enormous advances in the detection and treatment of breast cancer, it still remains the leading cancer diagnosis and has the second highest mortality rate. Thus, breast cancer research is a high priority for academics and clinicians alike. Based on previous research indicating the potential of nitric oxide (NO) and SMYD-3 inhibition, this work sought to expand upon these concepts and combine the two approaches. Both NO (from *S*-Nitrosoglutathione (GSNO)), termed Group 1, and a combination therapeutic, inhibitor-4 (SMYD-3 inhibitor) plus NO (from GSNO), termed Group 2, were evaluated for their efficacy on breast carcinoma cell lines MCF7 and MDA-MB-231, and the normal MCF10A breast cell line, using cellular viability, colony formation capacity, cytotoxicity, and cellular apoptosis analysis. These results indicated that, in Group 1, breast carcinoma lines MCF7 and MDA-MB-231, cells experienced a moderate reduction in cellular viability (~20–25%), a large reduction in colony formation capacity (~80–90%), a moderate increase in the relative number of dead cells, and a moderate increase in cellular apoptosis. Group 2 was significantly more impactful, with a ~50% knockdown in cellular viability, a 100% reduction in colony formation capacity, a large increase in the relative number of dead cells, and a large increase in cellular apoptosis. Additionally, Group 2 induced a very small impact on the normal MCF10A cell line. Cumulatively, this work revealed the exciting impact of this combination therapeutic, indicating its potential for clinical application and further research.

## 1. Introduction

Female breast cancer cases in the United States have been continually increasing over the past 20 years [1]. Fortunately, 5-year survival rates remain optimistic [2]. This is possible because of continued advancements in breast cancer detection and treatment options. In modern practice, the course of treatment is dependent upon three subcategories of breast cancer that are defined by the presence or absence of specific molecular markers for estrogen or progesterone receptors and human epidermal growth factor 2 (ERBB2; formerly HER2) [3,4]. Currently, chemotherapy, endocrine therapy, and ERBB2-targeted antibody or small molecule inhibitor therapy combined with chemotherapy are mainstays in breast cancer treatment [2,5,6,7,8,9]. Nevertheless, breast cancer far exceeds all other cancers in new cancer diagnoses and has the second highest mortality rate [1].

Enormous efforts are dedicated to developing new, effective therapeutic options for breast cancer. For example, several studies have highlighted the potential of nitric oxide (NO) [10,11,12,13,14]. However, the effect of nitric oxide on breast cancers is dichotomous: augmenting tumor growth and encouraging metastasis at low concentrations (nanomolar) [11,15] while promoting tumor apoptosis and cytostasis at high concentrations (micromolar) [11,12,13,14]. The ability to cause site-specific delivery and control release kinetics limits its practical application. Thus, various NO-delivery platforms have been investigated, such as NONOates, *N*-diazeniumdiolates, and *S*-Nitrosothiols (RSNOs) [10,11,12,13,14,15,16,17,18,19,20,21]. RSNOs present two major advantages over other delivery platforms: they allow prolonged NO release and naturally exist in the body [16,17,22].

Another therapeutic strategy focuses on SMYD (SET and MYND domain-containing) family proteins as molecular targets in cancer treatment [23,24,25]. In particular, SMYD3 is known to regulate cancer cell growth and proliferation [23,24]. Consequently, SMYD3 overexpression has been linked to increased proliferation, transformation, and metastasis of cancer cells [26,27]. Multiple studies highlight that when SMYD3 is inhibited, a substantial decrease in proliferative capacity of breast cell lines is observed [23,28,29].

**Figure 1:**
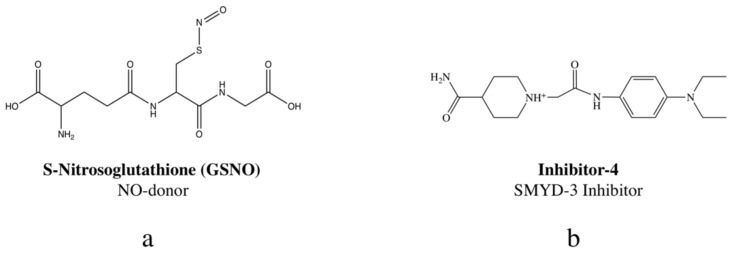
Structures of S-Nitrosoglutathione ((**a**) GSNO; NO-donor) and inhibitor-4 ((**b**) SMYD-3 inhibitor).

Herein, the novel combination of NO, delivered via *S*-Nitrosoglutathione (GSNO) (Figure 1a), and SMYD3 inhibition, with inhibitor-4 (Figure 1b), is investigated for anticancer potential against breast cancers. In previous studies, the RSNO, GSNO, was shown to exhibit tumoricidal characteristics [30,31,32]. Likewise, a separate study indicated that a SYMD3 inhibitor, inhibitor-4, demonstrated potent impacts on two breast cancer lines, MCF7 and MDA-MB-231 (both lines linked to overexpression of SMYD3) [29]. By combining the two for a combination therapeutic, GSNO and inhibitor-4, we sought to surpass the efficacy of each individual therapeutic on MCF7 and MDA-MB-231 breast cancer lines.

To evaluate and compare the efficacy of each individual therapeutic to the combination cocktail, cell viability, colony formation capacity, cytotoxicity, and cell apoptosis were examined. This manuscript contains the results pertaining to NO, delivered by 1 mM GSNO, hereafter described as Group 1, and the combination therapeutic, SMYD3 inhibitor (200 μM inhibitor-4) and NO (from 1 mM GSNO), hereafter described as Group 2. As previously mentioned, the findings pertaining to inhibitor-4 were investigated in a prior study [29]. Glutathione (GSH), the precursor to GSNO, was applied to cells as a functional control, highlighting NO as the active therapeutic (not GSNO). All of these results were considered in the final comparison.

## 2. Experimental Section

### 2.1. Materials

Dulbecco’s Modified Eagle’s Medium (DMEM), Eagle’s Minimum Essential Medium (EMEM), F-12 Medium, Gibco Horse Serum (New Zealand origin), Invitrogen Cholera Toxin Subunit B (Recombinant), Alexa Fluor 488 Conjugate, Lonza Walkersville MEGM Mammary Epithelial Cell Growth Medium SingleQuots Supplements and Growth Factors (Insulin, BPE, Hydrocortisone), Promega Caspase-Glo 3/7 Assay Kit, and Penicillin-Streptomycin Solution were obtained from Fisher Scientific (Hampton, NH, USA). EquaFETAL 100% Bovine Serum was purchased from Atlas Biologicals (Fort Collins, CO, USA). Reduced glutathione (GSH, High Purity), CellTiter-Blue Cell Viability Assay (CTB), and 3-(4,5-Dimethylthiazol-2-yl)-2,5-diphyltetrazolium bromide (MTT) were purchased from VWR International (Randor, PA, USA). Hydrochloric acid (HCl), EPA vials, Dead Cell Apoptosis Kit with Annexin V, FITC, and PI for flow cytometry, and Invitrogen ReadyProbes Cell Viability Imaging Kit were obtained from Thermo Fisher Scientific (Waltham, MA, USA). Dimethyl sulfoxide (DMSO) and Acetone (≥99.5%) were purchased from Sigma Aldrich (St. Louis, MO, USA). Sodium nitrate (99.999% NaNO_2_) was obtained from Alfa Aesar (Ward Hill, MA, USA). Trypsin/EDTA solution was purchased from American Type Culture Collections (Manassas, VA, USA). The breast cancer cell lines MCF7 and MDA-MB-231, as well as the healthy breast epithelial cell line used, MCF10A, were provided purchased from American Type Culture Collection (ATCC). SMYD-3 inhibitor (C_18_H_28_N_4_O_2_), aka inhibitor-4, was purchased from Enamine (Kyiv, Ukraine).

### 2.2. Synthesis of S-Nitrosoglutathione (GSNO)

*S*-Nitrosoglutathione (GSNO) was synthesized through a previously developed method. In brief, sodium nitrite (NaNO_2_) was added to a solution of reduced glutathione (GSH) in Millipore water and 2 M hydrochloric acid (HCl). The mixture was constantly stirred in an ice bath for 40 min. The solution was treated with acetone, followed by an additional 10 min of stirring in the ice bath (mixture turned a red color). The red solution was filtered for 10 min, first with gravity filtration for 10 min and then vacuum filtration for 3.5 h. After the GSNO precipitate was isolated, it was washed successively with ice-water and acetone. The remaining red filtrate solution was discarded while the solid pink powder (GSNO) was retained. The precipitate was analyzed by UV-Vis spectrophotometry at 335 nm to confirm <95 purity.

### 2.3. Cell Culture

Complete cell media (complete DMEM/EMEM) for both breast cancer cell lines consisted of 500 mL of DMEM/EMEM cell media supplemented with 10% total volume fetal bovine serum and 1% total volume penicillin-streptomycin. Complete cell media for healthy breast tissue cells, MCF10A, consisted of 500 mL of a 50/50 mixture of DMEM and F-12 media supplemented with 25 mL horse serum, 5 mL penicillin-streptomycin solution, 0.5 mL insulin, 2 mL BPE, 0.5 mL EGF, 0.5 mL hydrocortisone, and 50 µL of 0.1 mg/mL stock of cholera toxin. Initially, 1 mL containing 10^6^ cells was thawed for 1–2 min in a 37 °C water bath, added to a 15 mL centrifuge tube containing 9 mL of pre-warmed complete media, and centrifuged for 5 min at 4 °C, 2000× *g* rpm. Then, the supernatant was aspirated, and the remaining pellet was resuspended in 5 mL of complete media. This was added to T-25 cm^2^ flasks containing 5 mL of complete media. Cultures were incubated at 37 °C, 5% CO_2_ for at least 48 h before fresh complete media was provided every 24–72 h. Macroscopic observation and cell counting via hemocytometer were used to count and split cell cultures.

### 2.4. Cell Viability Assays

#### 2.4.1. Experimental Setup

In the cell viability assays, cells were plated at 100,000–200,000 cells/mL (MTT, CTB, respectively) for Group 1 and 500,000 cells/mL for Group 2 in 100 µL increments in 96-well plates and incubated at 37 °C, 5% CO_2_. The media was aspirated and discarded after 24 h. Group 1 positive control samples (PC; ≥4 samples) received 100 µL of complete media, samples received 100 µL of 1 mM GSNO (sample; ≥4 samples), and the functional control samples (GSH; ≥4 samples) received 100 µL of 1 mM GSH. The plate was incubated for 24 h. Group 2 positive control samples (PC; ≥4 samples) received 100 µL of complete media that was replaced after 24 h, samples received 100 µL of 200 µM inhibitor-4 for 24 h, followed by 24 h treatment with 100 µL of 1mM GSNO (sample; ≥4 samples), and the functional control samples (GSH; ≥4 samples) received 100 µL of 1 mM GSH that was replaced with media after 24 h. After another 24 h of incubation, on day 3, the media was aspirated, and each well was given 100 µL of complete media. The appropriate cell viability assay was then performed (Supplementary Materials, Excel File 3).

#### 2.4.2. Assay Procedures

##### MTT Assays

Following the outlined experimental setup, 10 µL of 12 mM MTT stock solution was added to each well. Plates were incubated for 3 h. Then, 50 µL of DMSO was added to each well. Plates were placed back in the incubator for 10 min to solubilize the MTT formazan. Finally, a microplate reader was used to measure absorbance values at 540 nm.

##### CTB Assays

Subsequent to the experimental setup above, 20 µL of CTB stock solution was added to each well. Then, plates were incubated for 3 h. Finally, a microplate reader was used to measure absorbance values at 570 nm and 600 nm.

In both assays, absorbance measurements were detected using a BioTek Synergy 2 Multi-Detection Microplate Reader. Data points represent the mean ± standard deviation (SD).

### 2.5. Colony Formation Assays

In the colony formation assays, cells were plated at 100,000 cells/mL (Group 1) and 500,000 cells/mL (Group 2) in 1 mL increments in 24-well plates and then incubated at 37 °C, 5% CO_2_ for 24 h in 24-well plates. The media was then aspirated from all wells and discarded. Group 1 positive control samples (PC; ≥4 samples) received 1 mL of complete media, samples received 1 mL of 1mM GSNO (sample; ≥4 samples), and the functional control samples (GSH; ≥4 samples) received 1 mL of 1 mM GSH. The plate was incubated for 24 h. Group 2 positive control samples (PC; ≥4 samples) received 1 mL of complete media that was replaced after 24 h, samples received 1 mL of 200 µM inhibitor-4 for 24 h, followed by 24 h treatment with 1 mL of 1mM GSNO (sample; ≥4 samples), and the functional control samples (GSH; ≥4 samples) received 1 mL of 1 mM GSH that was replaced with media after 24 h. After incubation, cells were harvested, counted, and then re-plated at 500 cells/mL in new 24-well plates. These plates were placed back into the incubator and observed every 24–72 h for up to three weeks via brightfield microscopy to determine the formation of colonies, defined as masses ≥ 50 cells.

Colony formation images were captured using an Invitrogen Cytation 7 Fluorescence Microscope (Carlsbad, CA, USA). Data points represent the mean ± standard deviation (SD).

### 2.6. LIVE/DEAD Assays

In the LIVE/DEAD assays, cells were plated at 100 µL increments of 100,000 cells/mL (Group 1) and 500,00 cells/mL (Group 2) in 96-well plates before 24 h of incubation at 37 °C, 5% CO_2_. Group 1 positive control samples (PC; ≥4 samples) received 100 µL of complete media, samples received 100 µL of 1mM GSNO (sample; ≥4 samples), and the functional control samples (GSH; ≥4 samples) received 100 µL of 1 mM GSH. The plate was incubated for 24 h. Group 2 positive control samples (PC; ≥4 samples) received 100 µL of complete media that was replaced after 24 h, samples received 100 µL of 200 µM inhibitor-4 for 24 h, followed by 24 h treatment with 100 µL of 1 mM GSNO (sample; ≥4 samples), and the functional control samples (GSH; ≥4 samples) received 100 µL of 1 mM GSH that was replaced with media after 24 h. Following another 24 h of incubation, 10 drops of both the Blue and Green ReadyProbes Cell Viability Imagine Kit stock solutions were added to 5 mL complete media. The media was aspirated from each well and 100 µL of the Blue/Green stock solution was added to each well before it was again incubated for 15 min. Imaging via fluorescent microscopy was used for qualitative comparison of the relative number of live cells versus dead cells in each well.

LIVE/DEAD images were captured using an Invitrogen Cytation 7 Fluorescence Microscope (Carlsbad, CA, USA). Live cells exhibited blue fluorescence while dead cells exhibited green fluorescence.

### 2.7. Cell Apoptosis Assays

#### 2.7.1. Caspase-Glo 3/7 Apoptosis Assay

In the Caspase-Glo 3/7 apoptosis assays, cells were plated at 100 µL increments of 100,000 cells/mL (Group 1) and 500,000 cells/mL (Group 2) in 96-well plates before 24 h of incubation at 37 °C, 5% CO_2_. Next, all of the media was aspirated and discarded before Group 1 positive control samples (PC; ≥4 samples) received 100 µL of complete media, samples received 100 µL of 1 mM GSNO (sample; ≥4 samples), and the functional control samples (GSH; ≥4 samples) received 100 µL of 1 mM GSH. The plate was incubated for 24 h. Group 2 positive control samples (PC; ≥4 samples) received 100 µL of complete media that was replaced after 24 h, samples received 100 µL of 200 µM inhibitor-4 for 24 h, followed by 24 h treatment with 100 µL of 1 mM GSNO (sample; ≥4 samples), and the functional control samples (GSH; ≥4 samples) received 100 µL of 1 mM GSH that was replaced with media after 24 h. Following another 24 h of incubation, all samples were given 100 µL Caspase-Glo 3/7 Reagent. Plates were incubated at room temperature for 2.5 h before luminescence of all samples was measured with a BioTek Synergy 2 Multi-Detection Microplate Reader (Winooski, VT, USA). Data points represent the mean ± standard deviation (SD).

#### 2.7.2. Annexin V/PI Apoptosis Assay

In the AnnexinV/PI apoptosis assays, cells were plated at 100 µL increments of 100,000 cells/mL (Group 1) and 500,000 cells/mL (Group 2) in 24-well plates before 24 h of incubation at 37 °C, 5% CO_2_. Next, all of the media was aspirated and discarded before Group 1 positive control samples (PC; ≥4 samples) received 100 µL of complete media, samples received 100 µL of 1 mM GSNO (sample; ≥4 samples), and the functional control samples (GSH; ≥4 samples) received 100 µL of 1 mM GSH. The plate was incubated for 24 h. Group 2 positive control samples (PC; ≥4 samples) received 100 µL of complete media that was replaced after 24 h, samples received 100 µL of 200 µM inhibitor-4 for 24 h, followed by 24 h treatment with 100 µL of 1mM GSNO (sample; ≥4 samples), and the functional control samples (GSH; ≥4 samples) received 100 µL of 1 mM GSH that was replaced with media after 24 h. Following another 24 h of incubation, all samples were harvested, washed with phosphate-buffered saline (PBS), re-centrifuged, and resuspended in 1X annexin-binding buffer. Cells were re-plated at 100 µL increments 1,000,000 cells/mL in 24-well plates. All wells received 5 µL FITC annexin V (Component A) and 1 µL of 100 µg/mL PI solution. Plates were incubated at room temperature for 15 min before 400 µL of annexin-binding buffer was added, mixed gently, and stored on ice. Cells were analyzed within 1 h by flow cytometry on a Cytek 4-laser Aurora instrument (Cytek, Fremont, CA, USA). A minimum of 3 × 10^4^ events was collected from each sample. Multivariate data were analyzed with SpectroFlo software (Cytek, Fremont, CA, USA). APC Annexin V−/PI−, APC Annexin V−/PI+, APC Annexin V+/PI−, APC or Annexin V+/PI+ represented viable, necrotic, early apoptotic, or late apoptotic cells, respectively.

### 2.8. Data Analysis and Statistics

All results were reported as an average and standard deviation with *n* > 5 for each group. One way-ANOVA was used to perform all statistical analysis and *p* < 0.05, 0.01, 0.001 (*, **, ***, respectively) defined statistically significant differences.

## 3. Results and Discussion

The overall goal of this study was to determine the relative efficacy of NO, delivered by GSNO only (Group 1), and the combination therapeutic, SMYD-3 inhibitor (inhibitor-4) and NO from GSNO (Group 2) on human breast carcinomas MCF7 and MDA-MB-231. The impact of inhibitor-4 alone was assessed in a separate study conducted by our collaborators, Alshiraihi et al. [29]. Various methods were used to determine efficacy, including cell viability assays (MTT and CTB), colony formation assays, LIVE/DEAD cytotoxicity assays, and cell apoptosis assays (Caspase-Glo 3/7 and Annexin V/PI). Additionally, normal MCF10A breast cells were evaluated in an identical manner to gauge the discriminatory capabilities of these treatment groups. In all assays, the sample groups consisted of positive control (PC) cells, sample (S) cells (Group 1 or Group 2), and functional control (GSH) cells.

### 3.1. Cell Viability Assays

Initially, the effects of two treatment groups, Group 1 and Group 2, were assessed for their impact on viability of MCF7 and MDA-MB-231 cells compared to normal breast cells, MCF10A. Both MTT and CTB cell viability assays showed a significant decrease in viability after the 24 h treatment period for each treatment group. Unfortunately, normal MCF10A cells treated with NO also experienced a similar decrease in viability when treated with Group 1. Optimistically, when treated with Group 2, normal MCF10A cells did not experience a more significant drop in viability. Importantly, the decrease in viability due to Group 2 was ~46–50%, which was significantly more pronounced than the NO-only Group 1 or the inhibitor-4-only group [29]. These results suggest that Group 2 significantly reduces the viability of breast carcinomas while retaining normal cell health.

#### 3.1.1. MTT Assay

Group 1

**Figure 2:**
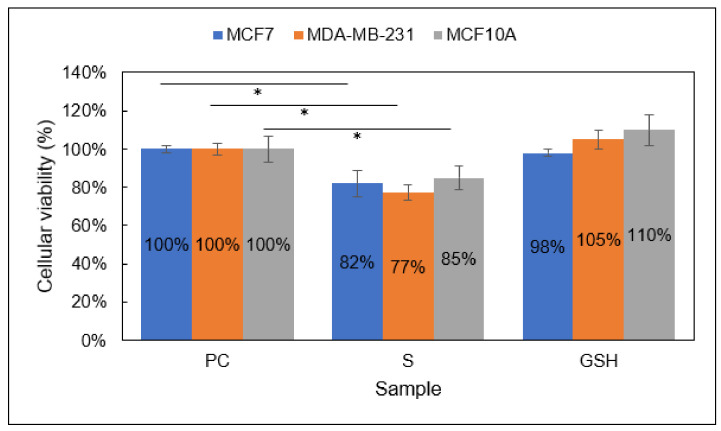
Cellular viability of MCF7 (blue), MDA-MB-231 (orange), and MCF10A (gray) cells analyzed with the MTT assay. Sample groups include: PC (untreated), S (Group 1), and GSH (functional control) cells. Data points represent the mean ± standard deviation. Statistical differences from the positive control are signified by * *p* < 0.05.

In Group 1, the MTT assay revealed a moderate decrease in MCF7, MDA-MB-231, and MCF10A cell viability (Figure 2). Explicitly, after the 24 h treatment period, the viability of MCF7, MDA-MB-231, and MCF10A S (NO-treated) cells was 82% ± 7%, 77% ± 4%, and 85% ± 6%, respectively. The viability of GSH-treated cells remained unchanged. Collectively, these data illustrate the potential of NO in breast carcinoma treatment. However, these data also reveal that NO (Group 1) cannot act as a standalone treatment and that normal breast cells are impacted, necessitating additional investigation.

Group 2

**Figure 3:**
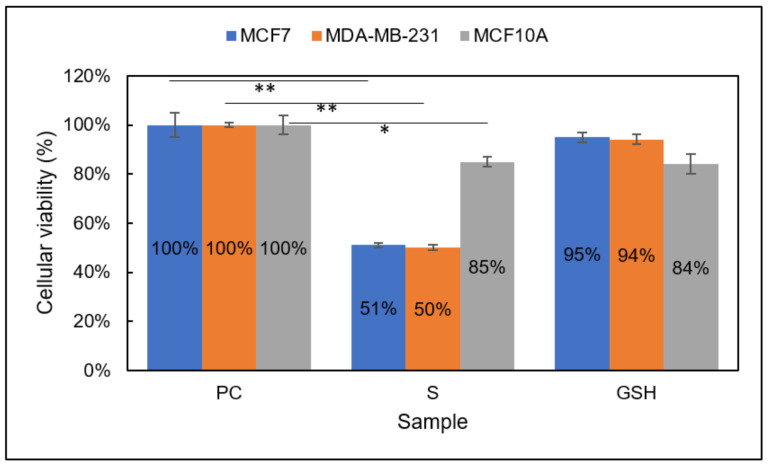
Cellular viability of MCF7 (blue), MDA-MB-231 (orange), and MCF10A (gray) cells analyzed using the MTT assay. Sample groups include: PC (untreated), S (Group 2), and GSH (functional control) cells. Data points represent the mean ± standard deviation. Statistical differences from the positive control are denoted by * *p* < 0.05, ** *p* < 0.01.

MTT assay results showed Group 2-treated MCF7 and MDA-MB-231 breast carcinomas experienced a significant reduction in cellular viability after 24 h (Figure 3). MCF7 and MDA-MB-231 S (Group 2) cells showed 51% ± 1% and 50% ± 1% viability, respectively. Additionally, 85% ± 2% of normal MCF10A cells retained viability after treatment. These data strongly indicate the increased efficacy of Group 2 on breast carcinomas versus NO (Group 1) alone or inhibitor-4 alone [29]. Individually, NO and inhibitor-4 alone reduced the cellular viability of both breast cancer lines by ~20–25% each, so the cumulative decrease displayed here, ~50%, was expected. It was not clear whether normal cells would experience the same reduction in viability. Group 1 cells did experience a ~15% reduction in viability when exposed to NO only (Figure 2). However, inhibitor-4-exposed cells did not experience a statistically significant reduction in viability. Fortunately, the Group 2 results indicated a similar, ~15%, reduction in viability to the Group 1 results.

#### 3.1.2. CTB Assay

Group 1

**Figure 4:**
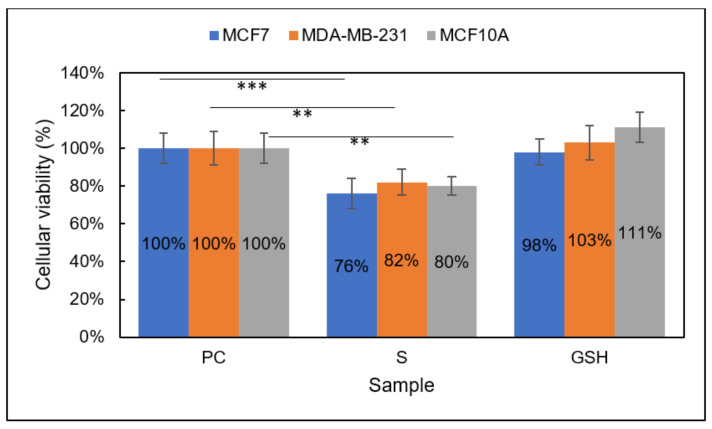
Cellular viability of MCF7 (blue), MDA-MB-231 (orange), and MCF10A (gray) cells analyzed using the CTB assay. Sample groups include: PC (untreated), S (Group 1), and GSH (functional control) cells analyzed using the CTB assay. Data points represent the mean ± standard deviation. Statistical differences from the positive control are indicated by ** *p* < 0.01, *** *p* < 0.001.

Comparable to the MTT assay results, the CTB assay revealed a decrease in MCF7, MDA-MB-231, and MCF10A cell viability in Group 1 (Figure 4). After the treatment period, the CTB assay showed S viability of MCF7, MDA-MB-231, and MCF10A cells to be 76% ± 8%, 82% ± 7%, and 80% ± 5%, respectively, after the 24 h treatment period. Again, these results echo those from the MTT assay results, showing a moderate decrease in viability of breast carcinomas as well as normal breast cells. Taken together, Group 1 does not show vast promise as a standalone treatment for breast carcinomas.

Group 2

**Figure 5:**
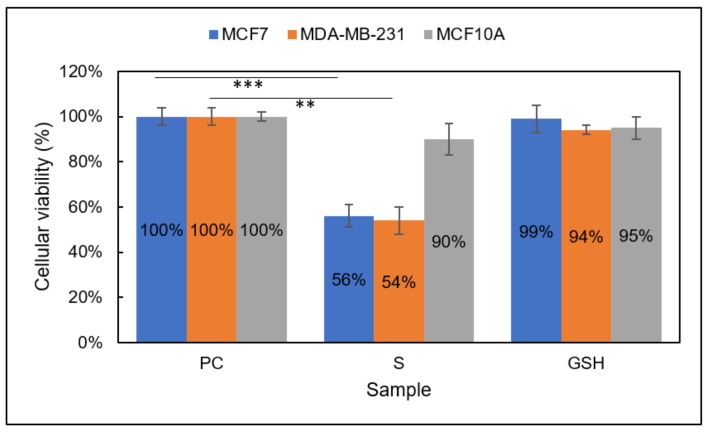
Cellular viability of MCF7 (blue), MDA-MB-231 (orange), and MCF10A (gray) cells analyzed using the CTB assay. Sample groups include: PC (untreated), S (Group 2), and GSH (functional control) cells analyzed with the CTB assay. Data points represent the mean ± standard deviation. Statistical differences from the positive control are represented by ** *p* < 0.01, *** *p* < 0.001.

Again, the CTB assay results here reiterate those of the MTT assay above, indicating significant reduction in both breast carcinoma lines and preserving the viability of the normal breast line in Group 2 (Figure 5). After 24 h treatment with each therapeutic consecutively, the viability of MCF7 and MDA-MB-231 S cells was 56% ± 5% and 54% ± 6%, respectively. The viability of normal MCF10A cells was 90 ± 7%. These data, along with the MTT data above, confirm the improved impact of Group 2 versus either therapeutic individually [29]. Collectively, the viability data collected prompted further investigation of the role of Group 1 and Group 2.

### 3.2. Colony Formation Assays

Clonogenic capacity is an important indicator of therapeutic efficacy. As such, MCF7, MDA-MB-231, and MCF10A cells were allowed to form colonies after 24 h treatment with each of the two treatment groups, Group 1 and Group 2. Both treatment groups significantly reduced colony formation (defined as masses of >50 cells) in both breast cancers (MCF7 and MDA-MB-231). Unfortunately, both groups also significantly reduced colony formation in the normal MCF10A cell line. Colony formation was further reduced in Group 2, but only by a small margin. Additionally, colony formation was completely halted in Group 2 for both breast cancer cell lines. These results suggested a potential drawback in the conceivable application of each treatment option. However, further investigation was deserved.

Group 1

**Figure 6:**
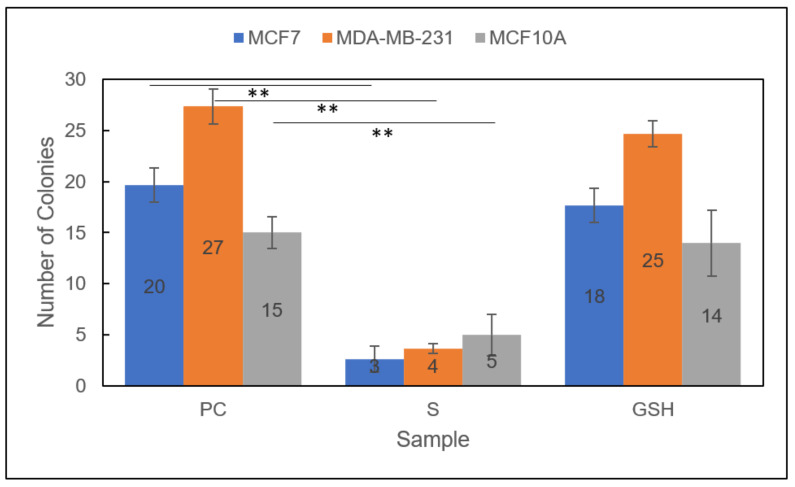
Colony formation capacity of MCF7 (blue), MDA-MB-231 (orange), and MCF10A (gray) cells. Sample groups include: PC (untreated), S (Group 1), and GSH (functional control) cells. Data points represent the mean ± standard deviation. Statistical differences from the positive control are shown by ** *p* < 0.01.

The colony formation capacity of both breast carcinoma lines, MCF7 and MDA-MB-231 cells, and the normal breast cell line, MCF10A, was decreased significantly after 24 h in Group 1 (Figure 6). The number of colonies formed for MCF7 cells decreased from 20 ± 2 for untreated PC cells to 3 ± 1 for S (Group 1) cells. Similarly, the number of colonies formed for MDA-MB-231 cells decreased from 27 ± 2 for untreated cells to 4 ± 1 for S cells. Additionally, the number of colonies formed after NO-exposure (Group 1) for MCF10A cells was 5 ± 2 for S cells versus 15 ± 2 for untreated PC cells. The reduction in colony formation capacity in the normal MCF10A line presented a potential drawback to this therapeutic approach. Thus, other indicators of therapeutic efficacy were explored.

Group 2

**Figure 7:**
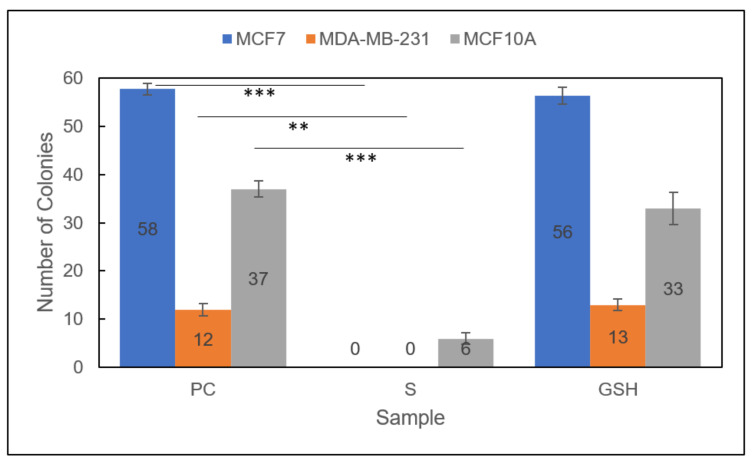
Colony formation capacity of treated *Group 2* MCF7 (blue), MDA-MB-231 (orange), and MCF10A (gray) cells. Sample groups include: untreated PC cells, S (Group 2) cells, and GSH-treated cells. Data points represent the mean ± standard deviation. Statistical differences from the positive control are indicated by ** *p* < 0.01, *** *p* < 0.001.

Promisingly, the colony formation capacity of all treated Group 2 cell lines was further reduced than in either therapeutic individually (Figure 7) [29]. Moreover, the clonogenic capacity of both breast carcinoma lines, MCF7 and MDA-MB-231 cells, was completely diminished to 0 after PC untreated cells formed 58 ± 1 and 12 ± 1, respectively. Again, the normal MCF10A cell line was also affected, reducing the number of colonies formed from 37 ± 2 to 6 ± 1. As with Group 1, these results indicated a potential drawback to the conceivable application of the Group 2 combinatorial therapeutic approach. Further studies would help determine the overall efficacy and prospective use of these therapeutic approaches for breast carcinomas.

### 3.3. LIVE/DEAD Assays

The next method employed to determine therapeutic potential of Group 1 and Group 2 was LIVE/DEAD cytotoxic analysis using fluorescence microscopy. Both breast carcinoma lines, MCF7 and MDA-MB-231 cells, as well as the normal breast cell line, MCF10A, were evaluated and exposed to both treatment groups. In both of the breast carcinoma lines, MCF7 and MDA-MB-231 cells, both treatment groups induced cell death, as shown by the green (dead)-stained cells below. These images also revealed that cell death did occur in MCF10A cells treated by both treatment groups; however, this occurred to a lesser extent than in the breast carcinoma cell lines. Collectively, these images further support the cellular viability and colony formation data analyzed above, highlighting the potential potency and discriminatory capability of the combination treatment approach (Group 2) on breast carcinomas.

Group 1

**Figure 8:**
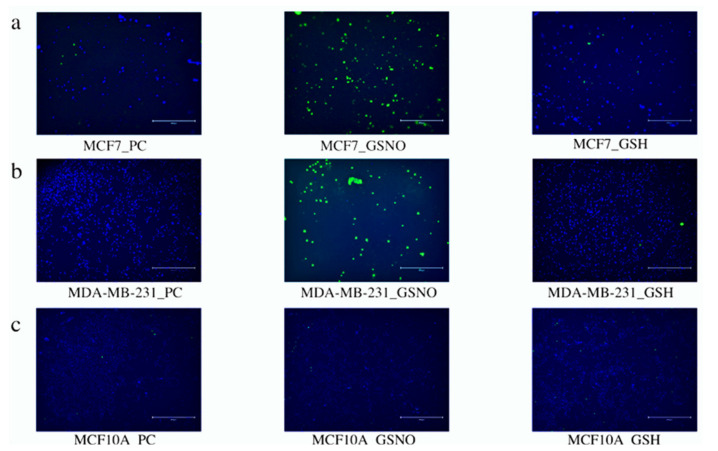
Live/dead cytotoxicity images for NO (Group 1) exposed MCF7 (**a**) and MDA-MB-231 (**b**) breast carcinoma cell lines as well as the normal breast cell line MCF10A (**c**). Images were captured using fluorescence microscopy. Live cells exhibited blue fluorescence and dead cells exhibited green fluorescence. For each cell line, the images on the left-hand side represent untreated PC cells, the images in the middle represent the S (Group 1) cells, and the images on the far right represent GSH-treated cells.

Optimistically, the LIVE/DEAD fluorescence microscopic analysis revealed that both breast carcinoma cell lines, MCF 7 and MDA-MB-231 cells, were significantly more impacted by Group 1 than normal MCF10A breast cells. This can be seen in the middle images in (Figure 8), where there is obviously more green (dead) staining in the top two images (MCF7 and MDA-MB-231 cells) than the bottom image (MCF10A). The untreated PC images and the GSH-treated images show almost exclusively blue (live) staining. These results encouraged further exploration of cell apoptosis to thoroughly investigate various facets of therapeutic efficacy.

Group 2

**Figure 9:**
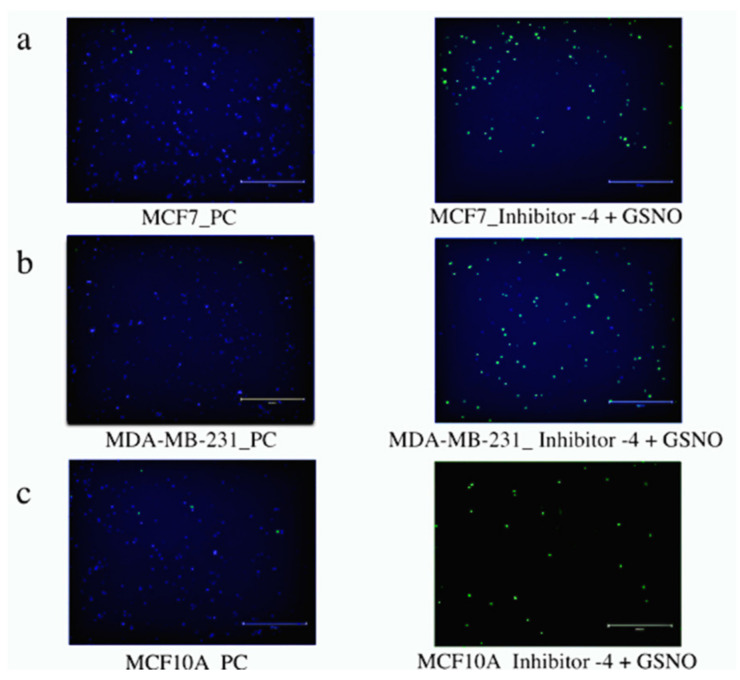
Live/dead cytotoxicity images for Group 2 MCF7 (**a**) and MDA-MB-231 (**b**) breast carcinoma cell lines as well as the normal breast cell line MCF10A (**c**). Images were captured using fluorescence microscopy. Live cells exhibited blue fluorescence while dead cells exhibited green fluorescence. For each cell line, the images on the left side represent untreated PC cells and the images on the right represent the S cells (Group 2).

Encouragingly, the LIVE/DEAD fluorescence microscopic analysis of Group 2 MCF7, MDA-MB-231, and MCF10A cells also reiterated the cell viability and colony formation results from above (Figure 9). Significantly more dead (green) cells are visible in the breast carcinoma lines, MCF7 and MDA-MB-231 cells, than in the normal breast cell line, MCF10A. Additionally, it is not obvious whether there are significant differences between these images and those of the cells exposed to Group 1 (Figure 8). Similarly, these results prompted further exploration of cell apoptosis.

### 3.4. Cell Apoptosis Assays

#### 3.4.1. Caspase-Glo 3/7 Apoptosis Assay

Finally, the last major indicator of therapeutic efficacy assessed in this work was the evaluation of cell apoptosis. The impact of both treatment groups, Group 1 and Group 2, was assessed for the induction of cell apoptosis of MCF7 and MDA-MB-231 breast carcinoma cell lines, as well as normal breast cell line, MCF10A. Two different methods were employed, including a Caspase-Glo 3/7 Assay, to probe for caspase 3/7-mediated induction of apoptosis and Annexin V/FITC and PI staining to display early/late apoptosis and necrosis. Overall, the results showed that Group 2 showed a significantly higher amount of cellular apoptosis in the breast carcinoma cell lines MCF7 (seen via Annexin V/FITC and PI staining) and MDA-MB-231 (seen via increased Caspase 3/7 activity and Annexin V/FITC and PI staining). Annexin V/FITC and PI staining revealed only a very slight increase in apoptosis in the normal MCF10A line. These results support and further reinforce the data collected above, emphasizing the promising potential for this combination therapeutic approach (Group 2) in breast carcinomas.

Group 1

**Figure 10:**
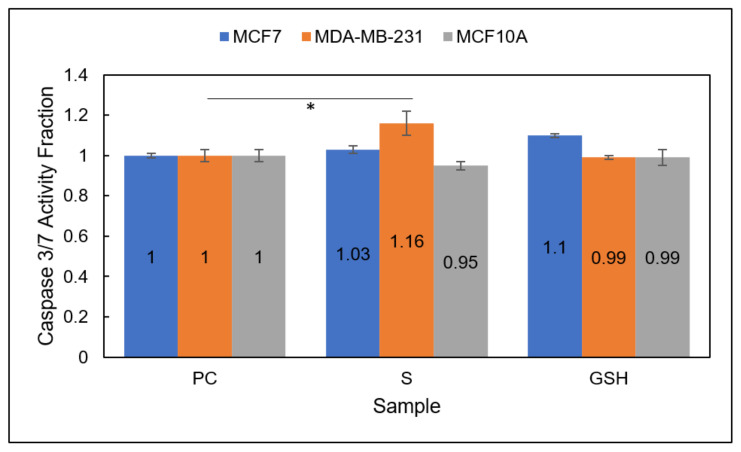
Caspase 3/7 activity fraction of NO (Group 1)-treated MCF7 (blue), MDA-MB-231 (orange), and MCF10A (gray) cells. Sample groups include: untreated PC cells, S (Group 1) cells, and GSH-treated cells. Data points represent the mean ± standard deviation. Statistically different values from the control are represented by * *p* < 0.05.

Caspase 3/7 activation was probed for all three cell lines, MCF7, MDA-MB-231, and MCF10A, using the Caspase-Glo 3/7 assay with luminescence detection for Group 1 (Figure 10). Caspase activity was increased slightly for one breast carcinoma line, MDA-MB-231, from 1 ± 0.03 for untreated PC cells to 1.16 ± 0.06 for S (Group 1) cells. Caspase 3/7 activity was not increased for the MCF7 breast carcinoma line. These results make sense because MDA-MB-231 cells are known to experience caspase-mediated induction of apoptosis, while MCF7 cells are known to experience apoptosis without caspase activation [33,34,35]. The normal MCF10A line also did not experience an increase in caspase 3/7 activity. This makes sense if very little apoptosis occurred. Thus, the results identified NO (Group 1) as a cytotoxic agent in MDA-MB-231 cells.

Group 2

**Figure 11:**
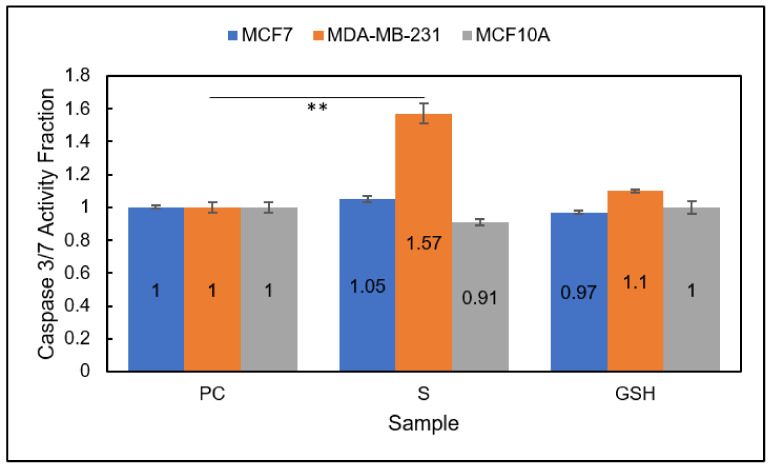
Caspase 3/7 activity fraction of MCF7 (blue), MDA-MB-231 (orange), and MCF10A (gray) cells detected using the Caspase-Glo 3/7 Assay. Sample groups include: untreated PC cells, S (Group 2) and GSH-treated cells. Data points represent the mean ± standard deviation. Statistically different values from the control are indicated by ** *p* < 0.01.

Caspase 3/7 activation was also evaluated for all three cell lines, MCF7, MDA-MB-231, and MCF10A, using the Caspase-Glo 3/7 assay with luminescence detection for Group 2 (Figure 11). Again, the only cell line to experience a change in caspase 3/7 activity was the breast carcinoma line, MDA-MB-231. Notably, the increase in caspase 3/7 activity was much more pronounced than when exposed to NO (Group 1) or inhibitor-4 alone [29]. After Group 2 MDA-MB-231 cells were treated, caspase 3/7 activity of treated cells was 1.57 ± 0.1 compared to untreated PC cells at 1 ± 0.03. Neither of the other two lines—the MCF7 breast carcinoma line and the normal cell line MCF10A—experienced a statistical change in caspase 3/7 activity. As previously mentioned, this makes sense because MCF7 and MCF10A cells experience caspase-independent apoptosis while MDA-MB-231 cells experience caspase-dependent apoptosis. These results emphasized the combinatorial treatment approach (Group 2) as a more potent apoptosis-inducing agent on MDA-MB-231 breast carcinomas than NO (Group 1)- or inhibitor-4 treated cells.

#### 3.4.2. Annexin V/PI Apoptosis Assay

**Figure 12:**
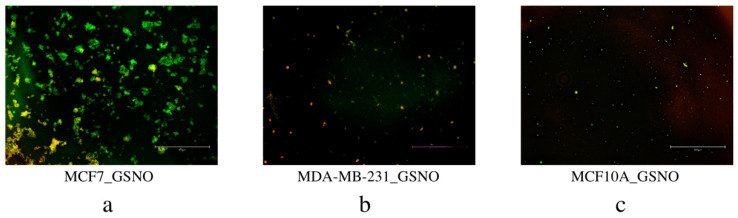
Detection of apoptosis in MCF7 (**a**), MDA-MB-231 (**b**), and MCF10A (**c**) cells by Annexin V/FITC and PI staining. Cells were treated with NO (Group 1) for 24 h, stained with Annexin V/FITC (green) and PI (red), and then imaged with fluorescence microscopy.

**Figure 13:**
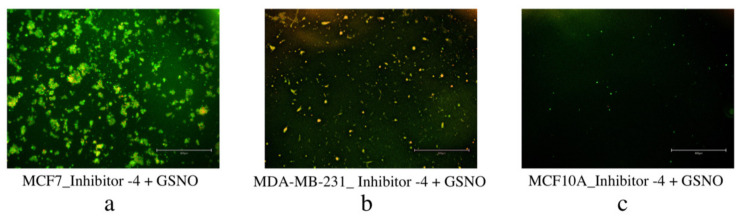
Detection of apoptosis in MCF7 (**a**), MDA-MB-231 (**b**), and MCF10A (**c**) cells by Annexin V/FITC and PI staining. Cells were treated with the combination therapeutic (Group 2), then stained with Annexin V/FITC (green) and PI (red) and imaged with fluorescence microscopy.

The second method employed to evaluate apoptosis and the final method to determine overall efficacy in NO (Group 1) and the combination therapeutic (Group 2) was Annexin V-FITC and PI staining with fluorescence microscopy. These data showed the occurrence of apoptosis (early or late stage) as well as necrosis in each sample group (PC, S, GSH) for all three cell lines, MCF7, MDA-MB-231, and MCF10A. Untreated PC cells and GSH-treated cells for all three did not show anything of note, so only the images of S cells are included below (Figure 12 and Figure 13). Overall, each treatment (Group 1 and Group 2) induced significant apoptosis and some necrosis in each of the two breast carcinoma lines, MCF7 and MDA-MB-231 cells. Only a very small amount of fluorescence corresponding to apoptosis was observed in MCF10A cells. These exciting results further reinforced the promising potential of NO (Group 1) and NO + inhibitor-4 combinations (Group 2) in breast carcinoma treatment.

Group 1

Apoptosis of NO (Group 1)-treated MCF7, MDA-MB-231, and MCF10A cells was analyzed using Annexin V-FITC and PI staining to observe early (Annexin V-FITC only, green) or late-stage apoptosis (both Annexin V-FITC and PI, green and red) as well as necrosis (PI only, red) (Figure 12). Obviously, both MCF7 and MDA-MB-231 breast cancer lines exhibit much more fluorescence, indicating a higher amount of apoptosis than normal MCF10A cells. Additionally, MCF7 cells mostly experienced early apoptosis while MDA-MB-231 cells mostly entered late apoptosis. These results identify Group 1 as an apoptosis-inducing agent in MCF7 and MDA-MB-231 cells.

Group 2

Similarly, MCF7, MDA-MB-231, and MCF10A cells consecutively exposed to the combination therapeutic (Group 2), were analyzed for apoptosis using Annexin V-FITC and PI staining (Figure 13). Again, early apoptosis (Annexin V-FITC only, green), late apoptosis (both Annexin V-FITC and PI, green and red), and necrosis (PI only, red) were observed for each line. Treated MCF7 and MDA-MB-231 breast carcinoma cells displayed significant fluorescence, indicating significant apoptosis in each line. Normal MCF10A cells displayed a very small amount of green fluorescence, indicating a small amount of early stage apoptosis. These results show that the combination treatment approach (Group 2) is a more potent treatment option than NO (Group 1) or inhibitor-4 alone. [29] Ultimately, Group 2 is highly efficacious in cell-line-based breast carcinoma research and deserves continued investigation.

## 4. Conclusions

Ultimately, these results highlight the Group 2 combination therapeutic, NO (from GSNO) + SMYD-3 inhibition (from inhibitor-4) as an extremely promising treatment for continued application in breast carcinoma treatment. In particular, this treatment shows discriminatory potential to breast carcinomas while imparting very few negative effects on normal breast cells. Future studies of both NO and NO combinations with SMYD-3 inhibition on breast cancers are necessary to determine their efficacy in clinical applications, such as 3D cell culture platforms and animal models.

## Data Availability

The data presented in this study is available within the article and supplementary materials.

## Supplementary Materials

The following are available online at https://www.mdpi.com/article/10.3390/diseases9040082/s1, Excel File 1: Apoptosis, Excel File 2: CF Results, Excel File 3: Viability Data.
